# Simultaneous bilateral laparoscopic cortical-sparing adrenalectomy for bilateral pheochromocytomas in multiple endocrine neoplasia type 2

**DOI:** 10.3389/fsurg.2022.1057821

**Published:** 2023-01-10

**Authors:** Xiao-Ping Qi, Bi-Jun Lian, Xu-Dong Fang, Fang Dong, Feng Li, Hang-Yang Jin, Ke Zhang, Kang-Er Wang, Yi Zhang

**Affiliations:** ^1^ Department of Oncologic and Urologic Surgery, The 903rd PLA Hospital, Hangzhou Medical College, Hangzhou, China; ^2^ Center for Radiation Oncology, Affiliated Hangzhou Cancer Hospital, Zhejiang University School of Medicine, Hangzhou, China; ^3^ Department of Urology, Affiliated Hospital of Hangzhou Normal University, Hangzhou, China; ^4^ Department of Urology, The Affiliated People's Hospital of Ningbo University, Ningbo, China

**Keywords:** multiple endocrine neoplasia type 2, pheochromocytoma, simultaneous bilateral adrenal-sparing adrenalectomy, laparoscopy, RET proto-oncogene

## Abstract

**Purpose:**

This study aimed to assess the feasibility of synchronous bilateral laparoscopic or open cortical-sparing adrenalectomy (SB-LCSA or SB-OCSA) for bilateral pheochromocytomas (bPHEOs) in multiple endocrine neoplasia type 2 (MEN2).

**Methods:**

Altogether, 31 patients (54.8% were women) were diagnosed with MEN2-related bPHEOs, and 29 of them underwent varying specific adrenalectomies. We systematically analyzed and evaluated their clinical profiles, mutation types, tumor histopathological features, and follow-up records.

**Results:**

All 31 patients with bPHEOs presented with *RET*-C634 (90.3%) and *RET*-M918T (9.7%) mutations, and the median age at initial presentation was 38 years (range, 23–78). bPHEOs were synchronous in 27 patients and metachronous in 4 (12.9%) patients. In total, 29 patients underwent initial cortical-sparing adrenalectomy (CSA) including 23 (79.3%) undergoing synchronous bilateral CSA (18 SB-LCSA and 5 SB-OCSA) and 6 (20.7%) undergoing metachronous CSA. SB-LCSA and synchronous surgery were associated with less bleeding volume and shorter length of hospital stay than SB-OCSA and metachronous surgery (all *P*’s < 0.05). Corticosteroid replacement treatment was necessary for 14 patients (45.2%) after bilateral CSA. During a median follow-up period of 7 years (range, 1.8–23), three of these patients (10.3%) had a recurrent disease that required reoperation.

**Conclusion:**

SB-LCSA is feasible for treating synchronous bPHEOs and should be recommended as a prioritized surgical approach.

## Introduction

Bilateral pheochromocytomas (bPHEOs) are extremely rare and may present either synchronously or metachronously. An overwhelming majority of these bPHEOs only develop as benign tumors, generally one of the clinical presentations of a syndrome, with 96% having a genetic predisposition (1–4). Approximately 89% of patients with bPHEOs develop multiple endocrine neoplasia type 2 (MEN2) caused by germline mutation of the *RET* (rearranged during transfection) proto-oncogene and von Hippel–Lindau (VHL) disease caused by the *VHL* gene (1). Other relatively less common genes associated with bPHEO include neurofibromatosis type 1 (*NF1*), succinate dehydrogenase (SDH) complex subunit D (*SDHD*), *SDHA*, *SDHB*, *SDHC*, SDH assembly factor 2, MYC-associated factor X, fumarate hydratase, transmembrane protein 127, and several recently reported genes that have not yet been rigorously evaluated, such as *KIF1B*, *SLC25A11*, and *MDH2* (1, 4–9). The *SDHB* mutation causing PHEO (paraganglioma) generally develops into a malignancy and metastasizes in ≥40% of affected patients (9, 14).

Currently, bPHEOs may be treated by a bilateral posterior retroperitoneal approach, laparotomy incision, or laparoscopic resection, while adequate preoperative treatment is administered with α-blockers. However, performing total adrenalectomy or cortical-sparing (adrenal-sparing) adrenalectomy (CSA) as the standard management of bPHEOs remains controversial. The main concerns on the risk of malignancy or future recurrences from remnants include the potential for the difficulty of reoperation and complications, the likelihood of corticosteroid independence to be balanced against the risks associated with chronically treated adrenal cortical insufficiency after CSA, and the inconsistent evidence of retrospective studies with small sample sizes. Recently, results of several international multicentre studies suggested laparoscopic/open operative CSA (LCSA/OCSA) as the successful surgical approach for patients with hereditary bPHEOs (1–3, 12). In particular, the bilateral LCSA (B-LCSA) approach should be considered a viable alternative option for patients requiring surgical intervention (1–4, 10–14).

However, regarding synchronous bilateral laparoscopic or open CSA (i.e., SB-LCSA or SB-OCSA) for bPHEOs associated with MEN2, less practical experience has been reported to date (1–4, 10–22). In the present study, 31 patients had bPHEOs originating from MEN2 in an ethnic Han Chinese cohort, of whom 29 underwent varying bilateral CSA including synchronous/metachronous LCSA/OCSA. Herein, their clinical presentation, genetic analysis, operative procedures, and surgical outcomes are described, and we further evaluated the safety and value of applying SB-LCSA for MEN2-related bPHEOs.

## Participants and methods

### Participants

This retrospective study analyzed prospectively collected data from November 1998 to April 2021. Of 258 patients (20.5%) belonging to 83 unrelated families, 53 presented with MEN2-related PHEOs (23–28). MEN2 was diagnosed based on genetic screening indicating germline mutations of the *RET* or clinical features and a clear family history for early cases. Subsequent *RET* testing was performed to confirm the diagnosis. In all individuals with MEN2, an initial clinical study, biological/imaging monitoring, and *RET* testing were conducted according to the following published criteria, as reported previously (23–28): each patient's clinical history/manifestations, physical examination, and biochemical tests, including plasma and 24-h urinary catecholamines (adrenaline, noradrenaline, and dopamine), vanillyl mandelic acid, and, after 2018, the addition of plasma metanephrines and normetanephrines. Imaging examinations included Doppler ultrasonography, computed tomography (CT), T2-weighted magnetic resonance imaging (MRI), and emission CT if indicated. The study protocol was approved by the Ethics Committee of the 903rd PLA Hospital, and written informed consent was obtained from all the participants or their legal guardians.

**Figure 1:**
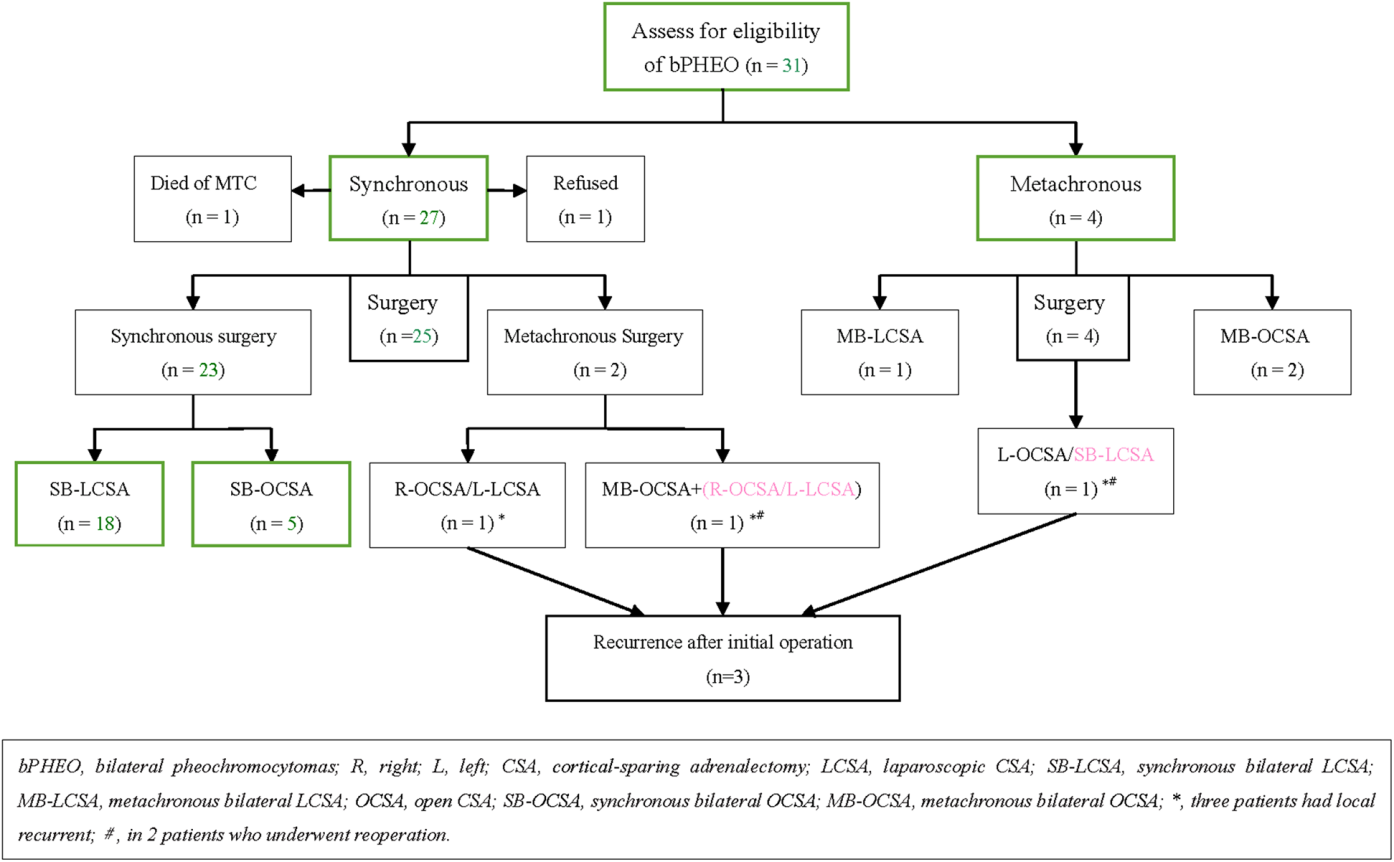
Study population and outcome by the type of operative intervention in those who had surgical intervention.

Overall, bPHEOs were diagnosed in 31 of 53 patients (58.5%) with MEN2-related PHEOs, depending on the biological/imaging examination and histopathological findings. In the 31 patients, of whom 29 (93.5%) received surgery, preoperative preparation was performed with adequate time to normalize blood pressure with alpha-blockers, and varying doses of phenoxybenzamine or terazosin were administered as the first choice to minimize perioperative cardiovascular complications. When blood pressure and heart rate control was poor, additional nifedipine or propranolol/metoprolol was provided; also, a high-sodium diet and fluids were provided preoperatively (10–14, 26, 29). On the morning of the operation day, phenoxybenzamine or terazosin was provided, while 100 mg of hydrocortisone was also infused intravenously. During the operation, the arterial blood pressure was monitored continuously, and 100–200 mg of hydrocortisone was infused intravenously. Of the remaining other two patients presenting MEN2A with synchronous bPHEOs, one (P26; male, 78 years) opted for treatment with terazosin over surgery and died of medullary thyroid carcinoma (MTC) in the lung and liver metastasis 2 years after the diagnosis of initial bPHEO and the other (P27; male, 25 years) declined further surgery for “watchful waiting” because of the absence of PHEO-related symptoms and a family factor (Table 1, Figure 1). Seven of these surgeries had OCAS *via* a laparotomy or lateral retroperitoneal open approach, which occurred before the year 2008. Subsequently, 19 patients had LCSA, and all the tumors were initially removed transperitoneally. The remaining three patients underwent at least an LCSA and/or OCAS as a hybrid surgery (Tables 1, 2).

**Table 1:** Demographic, clinical, and operative data of patients with MEN2-related bilateral pheochromocytomas.

Patient (No.)	Syn/Meta bPHEO (*n*, Syn %)	*RET* mutation	M/F	Age at operation (years)	Tumor size (cm), L/R	Surgery procedures	Multifocal (*n*, %)	Postoperative (*n*, %)		Follow-up^a^ (years)
								Steroid dependency	Recurrence	
P1	Syn	C634Y	M	56	6.0/5.5	SB-OCSA	Yes	—	—	17.5
P2		C634Y	M	38	2.0/7.0	SB-OCSA	Yes	—	—	15.0
P3		C634Y	M	42	3.2/4.6	SB-OCSA	Yes	Yes	—	16.5
P4		C634R	M	34	10.0/3.5	SB-OCSA	Yes	Yes	—	23.0
P5		C634Y	F	28	5.0/3.8	SB-OCSA	—	Yes	—	15.3
P6		C634Y	M	48	2.5/1.5	SB-LCSA	Yes	—	—	9.0
P7		C634Y	M	42	4.4/2.5	SB-LCSA	Yes	—	—	2.5
P8		C634G	F	45	4.0/4.0	SB-LCSA	Yes	—	—	5.5
P9		C634Y	M	42	2.2/2.8	SB-LCSA	—	—	—	7.0
P10		C634Y	F	38	3.3/3.8	SB-LCSA	Yes	—	—	6.0
P11		C634R	F	46	2.9/4.3	SB-LCSA	Yes	—	—	4.0
P12		C634Y	M	58	8.5/5.5	SB-LCSA	Yes	Yes	—	1.8
P13		C634Y	M	35	2.8/2.8	SB-LCSA	—	Yes	—	11.0
P14		C634Y	M	36	6.0/5.2	SB-LCSA	Yes	Yes	—	10.0
P15		C634Y	F	49	3.6/1.1	SB-LCSA	Yes	Yes	—	6.0
P16		C634Y	F	32	5.0/3.8	SB-LCSA	Yes	Yes	—	11.0
P17		M918T	F	23	6.0/5.0	SB-LCSA	Yes	Yes	—	3.5
P18		M918T	F	50	8.1/5.1	SB-LCSA	Yes	Yes	—	3.0
P19		C634G	F	52	3.0/3.5	SB-LCSA	Yes	—	—	3.8
P20		C634F	F	34	2.1/2.7	SB-LCSA	—	—	—	6.0
P21		C634Y	F	30	5.2/2.2	SB-LCSA	Yes	—	—	2.3
P22		C634S	F	37	1.2/3.5	SB-LCSA	—	—	—	5.0
P23		C634W	F	34	4.5/1.5	SB-LCSA	Yes	—	—	2.5
P24		C634Y	F	45 (two-step)	2.3/2.9	R-OCSA/l-LCSA	Yes	Yes	Yes	10.0
P25		C634R	F	24 (two-step)// 29/40	6.0/3.7	MB-OCSA// R-OCSA/l-LCSA/	Yes	Yes	Yes	15.3
P26		C634Y	M	78	3.0/3.4	Died	/	/	/	2.0
P27		M918T	M	25	1.7/1.8	Refused	/	/	/	0.3
P28	*Meta*	C634R	M	37/41	2.0/4.0	L-LCSA/R-LCSA	Yes	—	—	4.0
P29		C634Y	F	30/40	4.5/5.3	L-OCSA//SB-LCSA	Yes	Yes	Yes	7.0
P30		C634Y	F	27/44	7.5/11.0	R-OCSA/l-OCSA	Yes	Yes	—	20.2
P31		C634Y	M	44/49	4.3/4.5	R-OCSA/l-OCSA	Yes	—	—	16.0
Total	27/4 (87.1)	C634/M918T	14/17 (54.8^b^)	38^c^	4/3.8	29/31 (93.5)^d^	24/31 29 (82.8)	14/29 (48.3)	3/29 (10.7)	7.0 (1.8–23)^e^

Syn, synchronous; Meta, metachronous; bPHEO, bilateral pheochromocytoma; M, male; F, female; CSA, cortical-sparing adrenalectomy; LCSA, laparoscopic CSA; SB-LCSA, synchronous bilateral LCSA; MB-LCSA, metachronous bilateral LCSA; OCSA, open CSA; SB-OCSA, synchronous bilateral OCSA; MB-OCSA, metachronous bilateral OCSA. —, negative.

^a^ From the time of initial bilateral CSA to present.

^b^ F %.

^c^ Median age at initial diagnosis of bPHEO.

^d^ Number and proportion of patients who had operation.

^e^ Median of follow-up and time ranges except for two patients (P26 and P27) having no surgery.

**Table 2:** Characteristics of synchronous versus metachronous presentation of bilateral pheochromocytomas.

Variables	Synchronous PHEO^a^	Metachronous PHEO^a^	*P* value
Patient^b^, no. (%)	27 (87.1%)	4 (12.9%)	
Gender (male/female)	12/15	2/2	0.829
Mean age at diagnosis (years)	40.8 ± 12.0	43.5 ± 4.0	0.660
Median age at surgery (years)	38	42.5	
*RET* mutation (no.)	27	4	
C634F/G/Y/R/S/W (no.)	24	4	
M918T (no.)	3	0	
Adrenergic symptoms^c^			
Symptomatic	22	2	0.158
Asymptomatic	5	2	
Tumor size (cm)			
Mean	3.9 ± 1.9	5.4 ± 2.7	0.055
Median	3.5	4.5	
Tumor multicentric, no. (%)	20 (64.5%)	4 (12.9%)	0.248
Surgery procedures	25	4	
LCSA (no.)	18	1	
OCSA (no.)	5	2	
Hybrid (no.)	2	1	

PHEO, pheochromocytoma; CSA, cortical-sparing adrenalectomy; LCSA, laparoscopic CSA; OCSA, open CSA; hybrid, both have OCSA and LCSA.

^a^ Initial synchronous or metachronous PHEO.

^b^ Available data.

^c^ Including hypertension, palpitations/tachycardia, headaches, perspiration, etc.

Intraoperative blood loss volume was defined as the total amount of blood lost (from skin incision to the end of surgery). Length of hospital stay was defined as the length of postoperative stay in the department. Complications were classified using the modified Clavien–Dindo classification (CDC, grades I–V) (30), although operative time was not evaluated because of a relatively longer time span. Overall survival was assessed from the date of initial PHEO diagnosis to the time of the last follow-up.

### Surgical approach

Intravenous inhalation combined with general endotracheal anesthesia was used. SB-LCSA/metachronous B-LCSA (i.e., MB-LCSA), synchronous/metachronous bilateral OCSA (i.e., SB-OCSA or MB-OCSA), or hybrid LCSA/OCSA was performed (Figure 1). LCSAs were all performed transabdominally with the patient in supine 40°–70° laterally [placed either on the left or right (easy side) lateral supine position for the first CSA and then changed to the contralateral supine for the second procedure]. Left LCSA procedures were performed using the three-trocar technique, and in the right LCSA procedures, a fourth trocar was added for retraction of the liver when necessary (12, 21, 31). OCSAs were performed transperitoneally with the patient in supine or using the posterior retroperitoneal approach. In SB-OCSA cases, during the surgical procedure, the patients should remain in supine in contrast to that in MB-OCSA.

Combined with preoperative imaging information, careful exploration was performed to avoid leaving PHEO tumors and completely preserve the uninvolved adrenal tissues during the operation. After complete hemostasis of the operative wound, a gel sponge or absorbable hemostatic gauze was placed, and two drainage tubes were placed on both sides.

### RET screening using targeted sequencing

Briefly, peripheral blood genomic DNA samples obtained from at least 258 individuals from 83 individual families were prepared for targeted sequencing using an Illumina HiSeq 2000 Analyzer. The methods used for DNA target capture, enrichment, elution, and targeted sequencing were previously described (25, 27, 28). The targeted sequencing results were further validated by Sanger sequencing using an ABI 3700 Genetic Analyzer (PerkinElmer, Fremont, CA, USA).

### Statistical analysis

All data were analyzed using the IBM SPSS version 20.0 (SPSS Inc., Chicago, IL, USA). The frequency of occurrence, percentages, and comparisons of enumeration variables were assessed using the chi-square test (*χ*^2^) or Fisher's exact test and Student's *t*-test for comparison between independent treatment groups. Significance was set at *P* < 0.05.

## Results

### Clinical and diagnostic data

**Figure 2:**
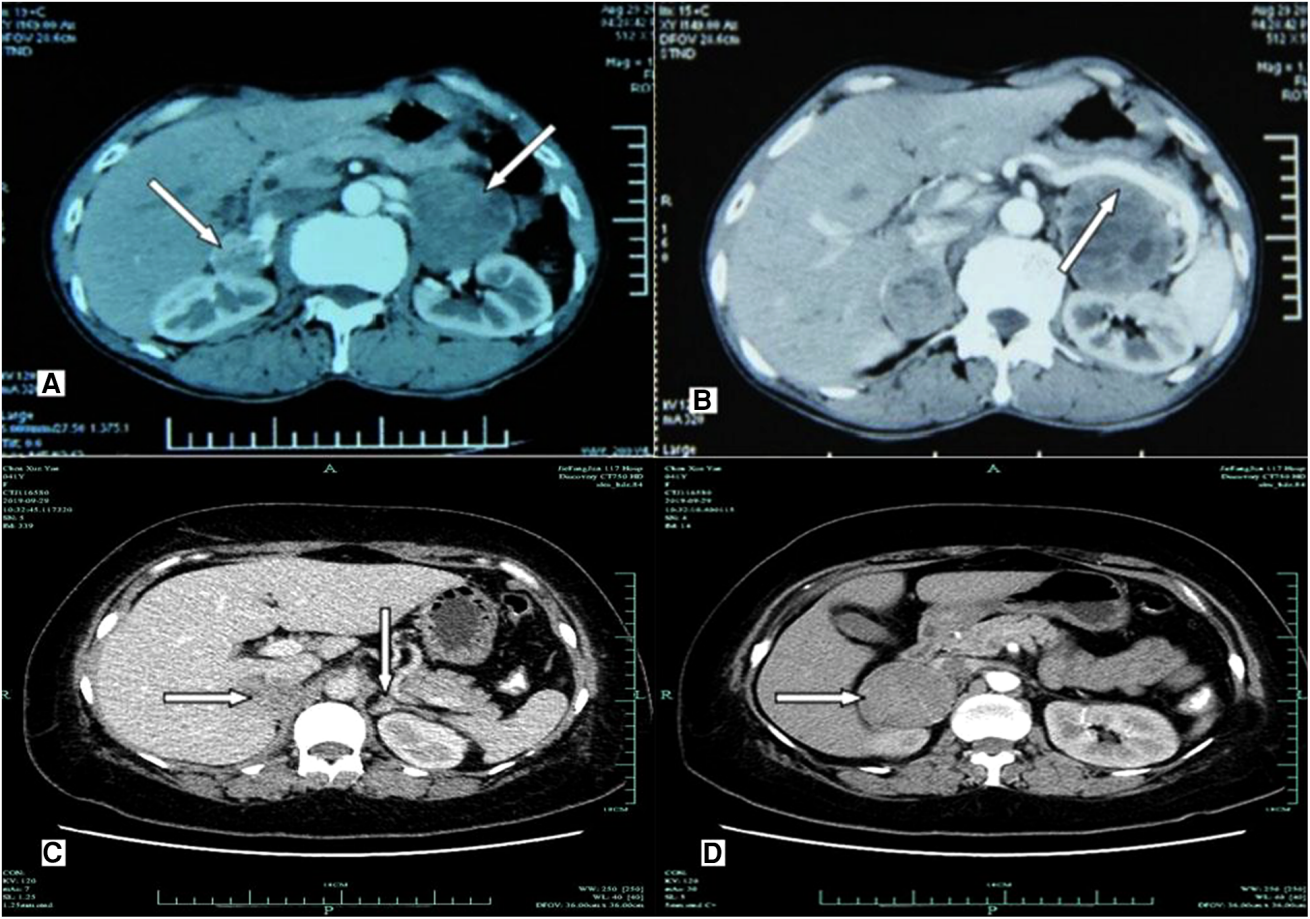
Imaging examination of computerized tomography scanning using ultravist 300 disclosed hypoechoic nodules in bilateral adrenal glands. (**A,B**) A 36-year-old male patient (P14; *RET*-C634Y) presenting with primary bilateral multifocal pheochromocytoma. (**A**) CT scan showing irregular enhancement of multiple nodules with intact capsule in both adrenal glands. (**B**) Contrast-enhanced CT imaging revealing inhomogeneous nodules with well-defined and heterogeneous enhancement in both adrenal glands (white arrows). The maximum diameter of tumors was 6.0 cm × 5.3 cm × 4.8 cm in the left, and the maximum diameter was 5.2 cm × 4.5 cm × 3.0 cm in the right, while having multiple low density lesions with no obvious enhancement, respectively. No areas of obvious necrosis or hemorrhage were observed. The splenic artery and vein showed compression and displacement (white arrow). (**C,D**) A 40-year-old female patient (P29; *RET*-C634Y) having bilateral pheochromocytoma. (**C**) Contrast-enhanced CT scanning showing that two primary inhomogeneous nodular and heterogeneous mild/moderate enhancement in the right adrenal (white arrows). A nodule (1.2 cm × 0.8 cm × 0.6 cm) with equal density in the left adrenal gland was enhanced to the same extent as the Normal adrenal tissue (white arrows), as possibility of recurrence of pheochromocytoma. (**D**) Larger one with clear boundary in the right adrenal was about 5.3 cm × 4.3 cm × 3.0 cm in diameter (white arrows).

Of all 31 patients with bPHEOs, 17 (54.8%) were women (Table 1). The median age at initial diagnosis of bPHEO was 38 years (range, 23–78). The median tumor size was 3.8 cm (range, 1.1–11.0), and the tumor was >5.0 cm in 38.7% of the cases (Figures 2A–D). Twenty-seven patients (87.1%) presented with initial synchronous PHEOs, and four patients (12.9%) had initial metachronous PHEOs; the incidence of a contralateral PHEO was 4, 5, 10, and 17 years after the first diagnosis/surgery of a unilateral PHEO (mean, 9 years). However, the mean age at diagnosis was not significantly different between patients with synchronous and those with metachronous PHEOs [(40.8 ± 12.0) vs. (43.5 ± 4.0) years, *P* = 0.660)], although patients with metachronous PHEOs may have an older median age by >4.5 years than those with synchronous PHEOs (Tables 1–3). Additionally, at the time of diagnosis of initial PHEOs, 7 patients (22.6%) were asymptomatic, and 24 (77.4%) presented with adrenergic symptoms, including hypertension (*n* = 24), headache (*n* = 9), palpitations/tachycardia, (*n* = 7), perspiration (*n* = 2), and paroxysms (*n* = 12). Among 25 patients (80.6%) who had available biochemical data, 14 (56%) presented with elevated catecholamines/MNs in plasma or urine. The diagnosis was made according to clinical features suggestive of PHEOs in 20 patients (64.5%) and according to mutations in 11 others (4 also had clinical features of the disease) (35.5%), which revealed that the mean size of a PHEO was 5.7 ± 2.0 cm in the patients discovered due to symptoms, which was larger than that of a PHEO detected through mutation-based screening (mean size: 3.2 ± 1.5 cm) (*P* = 0.003) (data not shown). The characteristics of synchronous vs. metachronous bPHEOs in the 31 patients are summarized in Tables 1, 2, respectively.

**Table 3:** Presence of perioperation and postoperation in synchronous/metachronous surgery.

Variables	Synchronous surgery	Metachronous surgery	*P* value
Patient (no.)	23 (79.3%)	6 (20.7%)	
Gender (male/female)	10/13	2/4	0.653
Mean age at surgery (years)	40.4 ± 9.0	37.5 ± 8.1	0.360
Median age at surgery (years)	38	40	
Tumor size (cm)			
Mean	4.1 ± 1.9	4.8 ± 2.5	0.245
Median	3.8	4.4	
LCSA/OCSA (no.)	36/10	6/9	0.005
Times of recovery (days)	19.5 ± 4.2	40.2 ± 8.9	0.000
Length of hospitalization (days)	11.4 ± 4.5	20.3 ± 8.4	0.016
Postoperative			
Complications^a^	0	0	
Steroid replacement	10	4	0.313
Recurrence, *n* (%)	0	3	0.000
Metastasis, *n* (%)	0	0	
Follow-up (years)	7.8 ± 5.6	10.0 ± 4.8	0.381

B-LCSA/B-OCSA, bilateral laparoscopic cortical-sparing adrenalectomy/bilateral open cortical-sparing adrenalectomy.

^a^ Clavien–Dindo classification, ≥grade II.

Moreover, all 31 patients presenting with bPHEOs were only associated with 11 and 16 exons of *RET*, predominantly in exon 11 mutation, and 28 participants (90.3%) harboring *RET-*C634F/G/R/S/W/Y mutations, followed by a C634Y (61.3%, 19/31), C634R (12.9%, 4/31), C634W (3.2%, 1/31), C634G (6.5%, 2/31), C634F (3.2%, 1/31), and C634S (3.2%, 1/31) and affected by MEN2A, belonged to 14 unrelated families. The three other participants (9.7%) carrying *RET-*M918T in exon 16 belonged to three different MEN2B families (Tables 1, 2). The mean age at bPHEO diagnosis was not significantly different between patients with C634 and those with M918T mutations [40.9 ± 11.4 vs. 32.7 ± 15.0 years; *P* = 0.256). Additionally, the mean tumor size was not significantly different (4.0 ± 2.0 vs. 4.6 ± 2.5 cm; *P* = 0.502), although those with *RET-*M918T were relatively younger by 8 years and had a tumor size that was approximately larger by 0.6 cm (Table 1). However, the diagnosis of initial PHEOs was also made after the diagnosis of MTC in 18 cases (58.1%), simultaneously in 5 cases (16.1%), and prior to the diagnosis of MTC in the 8 remaining cases (25.8%). The diagnosis of bPHEOs in all 31 patients was MTC (100%), and the youngest patient, aged 23 years, was diagnosed with synchronous PHEOs and presented with *RET-*M918T.

### Surgical procedures

Except for two patients (P26 and P27) who did not receive surgery, each of the other 29 patients (93.5%) underwent varying bilateral CSA. In 23 patients (79.3%), initial CSA was simultaneously performed on both sides, of whom 18 with SB-LCSA and 5 with SB-OCSA received the same anesthesia; whereas metachronous bilateral CSA was recorded in 6 cases (20.7%), including 1 with MB-LCSA, 2 with MB-OCSA, and 3 with LCSA/OCSA hybrid surgery (Table 1, Figure 1). Subsequently, dissection and ligation of the central adrenal vein were performed during the operation (Tables 1, 2). In particular, the surgical approach was initial LCSA in 19 cases (SB-LCSA in 18 participants with synchronous PHEOs and MB-LCSA in 1 patient with metachronous PHEOs). The LCSA approach was performed transabdominally and used for these 19 patients. OCSA was initially performed in 7 patients (5 SB-OCSA in 5 patients with synchronous PHEOs and 2 MB-OCSA in 2 patients with metachronous PHEOs), with 5 patients in the transperitoneal supine position and 2 patients in the posterior retroperitoneal position (Tables 1, 2; Figure 1). For the remaining three other individuals, one (P24) presented with synchronous bPHEOs, one had OCSA (right) and LCSA (left) interval 3 months (two-step surgery) in 2012, and the other (P29) who had initial unilateral PHEOs underwent initial OCSA (left) retroperitoneally in 2009. In 2020, due to recurrences of the left and newly developed right PHEOs (Figures 2C,D), an SB-LCSA (second surgery) was performed. Unfortunately, the third patient (P25) had synchronous PHEOs initially, underwent MB-OCSA with a 1-month interval in 2002, and underwent a second right OCSA or left LCSA in 2007 and 2018, respectively, due to recurrences of the right or left PHEOs. In total, 61 adrenal operations (30 right and 31 left) were performed using the LCSA (*n* = 42) or OCSA (*n* = 19), including the LCSA/OCAS hybrid surgical procedures (*n* = 3) (Tables 1–3, Figure 1).

The perioperative information including preoperative, intraoperative, and postoperative variables was compared between synchronous and metachronous CSA. Synchronous CSA had a significantly less recurrence rate and shorter length of hospital stay than metachronous CSA (Table 3). SB-LCSA had significantly less blood loss and a shorter length of hospital stay than SB-OCSA or SB-OCSA/hybrid surgery (Table 4; the latter data not shown). Meanwhile, the differences in surgical parameters were not significant between synchronous and metachronous CSA or between SB-LCSA and SB-OCSA (Tables 3, 4).

**Table 4:** Factors related to perioperation and postoperation in simultaneous bilateral laparoscopic or open surgery.

Variables	SB-LCSA	SB-OCSA	*P* value
Patient (no.)	18 (78.3%)	5 (21.7%)	
Gender (male/female)	6/12	4/1	0.062
Mean age at surgery (years)	40.6 ± 8.9	39.6 ± 10.5	0.831
BMI (kg/m^2^)	21.8 ± 2.7	22.0 ± 1.7	0.906
Tumor size (cm)			
Mean	3.8 ± 1.7	5.1 ± 2.3	0.060
Median	3.5	4.8	
ASA score (I/II/III/IV/V)	6/11/1/0/0	0/5/0/0/0	
Blood loss (ml)	75.0 ± 27.4	242.0 ± 100.8	0.003
Length of hospitalization (days)	10.0 ± 2.9	13.2 ± 1.6	0.046
Postoperative			
Complications^a^	0	0	
Steroid replacement, *n* (%)	7 (38.9%)	3 (60%)	0.397
Recurrence, *n* (%)	0	0	
Follow-up (mean, years)	5.3 ± 3.2	10.6 ± 5.4	0.000

CSA, cortical-sparing adrenalectomy; SB-LCSA, synchronous bilateral laparoscopic CSA; SB-OCSA, synchronous bilateral open CSA; hybrid, laparoscopic and open CSA.

^a^ Clavien–Dindo classification, ≥grade II.

Postoperatively, all 29 participants had histopathology verifying bPHEOs, of whom 24 patients (82.8%) had initial multicentric tumors, which are primarily noted in MEN2A (*n* = 22) and MEN2B (*n* = 2), and on the left in 19, right in 17, and bilateral multifocal in 12 patients (Table 1). The highest number of PHEOs was 9, of which 4 occurred on the left and 5 on the right, in a 56-year-old patient with C634Y mutation (P1; Table 1). No conversion to an open procedure was necessary. No serious intraoperative and postoperative complications (CDC, ≥grade II) were recorded. Laparoscopic manipulation and surgical removal of the PHEO resulted in eight peaks of hypertension [systolic blood pressure (SBP) > 180 mmHg], associated in four cases with bouts of sinus tachycardia and in two low peaks of hypotension (SBP < 60 mmHg) despite initial ligation of the central adrenal vein.

### Outcomes

Patients were all followed up for a median of 7 years (range, 1.8–23) from the time of initial CSA; 14 (48.3%) of them required lifelong steroid [glucocorticoid (prednisone or hydrocortisone) and mineralocorticoid] replacement (Table 1), and 2 (6.9%) of them had transient adrenal insufficiency and oversupplementation during the adjustment of drug dosage within 3 and 6 months postoperatively, although none developed the risk of Addisonian crisis.

The presence of recurrent PHEOs was diagnosed in three patients (10.7%; P29, P25, and P24), of whom one (P29) had a recurrence or developed contralateral PHEOs at 10 years postoperatively, and the other (P25) had bilateral recurrences at 4 and 16 years after the first operation; two underwent reoperation with no postoperative complications. Additionally, one patient (P24) had unilateral recurrence (tumor size, 1.5 cm) 3 years after MB-LCSA but declined surgery (Table 1). The former patient (P29) was diagnosed with recurrence due to adrenal symptoms, and the latter two patients (P25 and P24) had recurrence identified during re-examinations, with no lateral predisposition (two left adrenal beds and two right adrenal beds). The three patients with MEN2A had a mean time of recurrence of approximately 8 years (range, 3–16). Moreover, none of the tumors were malignant.

## Discussion

To the best of our knowledge, the present study is the first to detail the clinical presentation, management, and outcomes of patients with MEN2-related bPHEOs in an ethnic Han Chinese cohort. We identified bilateral disease in approximately 59% of the patients with PHEOs, which is similar to that previously reported by Castinetti et al. (2), who identified it in 61% of their cases. In this series, the bilateral disease was only associated with *RET-*C634 mutations, such as MEN2A (90%), *RET-*M918T, and MEN2B (10%), although the absence of other *RET* mutations demonstrated a relatively single mutation genotype (2, 13, 21, 32) (Tables 1, 2). Our finding also revealed that the bPHEO was exclusively benign, synchronously involved in both adrenal glands in approximately 80%, diagnosed at an asymptomatic stage in approximately 23%, and had a mean tumor size of 3.8 cm, whereas those observed in patients with PHEOs identified through mutation-based screening were less than symptomatic PHEOs (*P* = 0.003) (10, 32). In approximately 83% of multifocal cases, the affected patients mostly received the initial operation at <40 years of age (2, 3, 10, 13). However, the need for an optimal surgical procedure to manage these patients should still be highlighted regardless of whether PHEO-related deaths rarely occur. By contrast, most previous studies mainly focused on a more common cause of death and outcomes of MTC in these patients (2, 3, 10, 13). Thus, the use of prophylactic thyroidectomy in genotype-specific age or the extent of thyroidectomy based on genotype and serum calcitonin levels had become routine and the recommended formal practice guidelines (1–4, 9–13, 23–26, 33, 34).

By contrast, the second major component of MEN2-related PHEOs could be treated by laparoscopic excision based on an already established conventional procedure, which should be removed prior to surgery for either MTC or hyperparathyroidism (13, 14, 34). In the last two decades, laparoscopic bilateral adrenalectomy for bPHEOs demonstrated a sharp reduction of intraoperative hemodynamic instability, providing an equal opportunity for treating hypertension, less intraoperative blood loss, and lower overall complication rates while also causing a faster and better postoperative recovery and a better cosmetic result than the open approach (11–14, 20, 35, 36). In this study, similar results of less bleeding volume and shorter length of hospital stay were also revealed, which are possible with LCSA compared with those in OCSA or hybrid surgery (all *P*’s < 0.05; Tables 1–4 and Figure 1). The CSA approach for treating PHEOs should only be considered an alternative procedure or a relatively weak recommendation and not the established routine MEN2 practice guidelines (11–14, 34, 38, 39). The main concerns include the risk of remnant recurrences, reoperation, metastases, and the likelihood of corticosteroid independence after CSA. Nevertheless, CSA as a feasible surgical approach for unilateral/bilateral PHEOs in MEN2 patients was still performed by numerous clinicians (1–4, 10, 12, 15–23, 26, 33, 37–40). A recent multicenter study of 563 patients with MEN2-related PHEOs, which includes some patients from our cohort, has demonstrated that the incidence of malignant disease was <1%; bPHEO with CSA in one or two operated glands associated with a recurrence was 5% of 114 patients, of whom 57% were not steroid-dependent at a median of 9.5 years (range, 1–28) postoperatively (2). Another multicenter study of bPHEOs (*n* = 625) in 505 of 526 tested patients (96%) with germline mutations has detected that the majority of patients had *RET* mutations rather than *VHL* or other gene mutations (282 vs. 184 vs. 39, respectively) and that CSA was associated with a recurrence in 13% and malignant disease in 2% of patients at a follow-up of a median of 8 years (interquartile range, 4–17) (1). In this series, 29 patients underwent LCSA or OCSA to preserve most of the uninvolved adrenals using PHEO enucleation or subtotal adrenalectomy (CSA) with as much as possible rim (0.5–1.0 cm) of the normal adrenal tissue. Postoperatively, approximately 48% of these patients still required lifetime steroid replacement, and two of them (6.9%) suffered transient complications of steroid dosage. Meanwhile, approximately 10% of these patients experienced tumor recurrence, demonstrating that real recurrences are typically identified within 8 years (range, 3–16) or later after CSA, without metastatic or PHEO-related deaths (0%) (Tables 1, 3, 4; Figure 1). Evidently, nearly total adrenalectomy can be inevitable when a large tumor (such as P30) is in an unfavorable location, when multifocal tumors (P24) are present, or when reoperation for recurrent PHEOs (P25, P29) is necessary by the laparoscopic procedure. However, interestingly, patients (P1) with nine small multifocal tumors underwent PHEO enucleation and did not require steroid replacement postoperatively in the 17.5 years of follow-up (Table 1). Enucleation may be more beneficial for preserving vascularized adrenal cortical tissue/function than subtotal adrenalectomy as it preserves at least 10%–15% of one remnant of properly vascularized adrenal cortical tissue and may offer adrenal stress capacity (22, 31, 36). However, a long-term follow-up of at least 10 years is still necessary for all these patients because of persisting disease due to the risk of recurrent PHEOs of approximately 20% within 20 years after CSA (10, 11, 13). Nevertheless, the LCSA or OCSA approach did not decrease survival and may offer excellent oncologic and functional outcomes. Particularly, LCSA [including robotic surgery (37)] is a safe and effective surgical management for treating bilateral and/or multifocal PHEOs, especially for tumors <6 cm in MEN2. As for experienced surgeons, LCSA is also feasible for tumors >6 cm (36). The tran-peritoneal approach can provide more space and clearer anatomical structure, thus making it more suitable for treating large PHEOs. Thus, LCSA should be routinely recommended for bilateral and/or multifocal PHEOs (1–4, 10, 15–23, 26, 33, 35–40). Robot-assisted CSA has also been identified as an effective technique for the management of PHEOs (37). An important advantage of the robotic platform is the articulation of the instruments, which offers seven degrees of freedom, thus facilitating circumferential dissection of the tumor (41). Drawbacks of this approach mainly include a low penetration rate and increased cost (41). The robotic techniques have been demonstrated to be safe and feasible in several retrospective and prospective studies (37, 41). However, data regarding robotic CSA are derived only from small series, with no direct comparison between robotic and laparoscopic partial adrenalectomy. Nevertheless, robotic partial adrenalectomy represents an alternative option for partial adrenalectomy for experienced surgeons.

Additionally, treating patients with synchronous bPHEOs can be challenging, and no uniform standard surgical approach (for neither synchronous nor metachronous) has been established. Following laparoscopic device innovation, the accumulation of sufficient experience, and proficient surgical skills, synchronous surgery including SB-LCSA was increasingly used in clinical practice for treating these bPHEOs (10, 15–23, 41). Walz et al. (16) have reported that 15 patients with bPHEOs (average tumor size of 4.6 cm; 2 cases of recurrent PHEOs on one side) underwent synchronous bilateral laparoscopic adrenalectomy, in which 14 (93.3%) bilateral tumors were removed under the same anesthesia. In another case, the procedures were split due to cardiac arrhythmias during laparoscopic removal of a 12-cm right-sided PHEO, and the contralateral 3-cm tumor was extirpated 5 weeks later retroperitoneoscopically. Kittah et al. (10) have reported that of 75 patients (98.7%) with synchronous PHEOs (41 MEN2, 13 VHL, 7 NF1, and 14 other PHEOs; the median tumor size of 3.0 cm), 74 underwent a synchronous bilateral adrenalectomy and synchronous bilateral CSA was successfully performed in 18 (24%) of them. Nine (44.4%) of the 18 patients required steroid replacement, 3 (16.7%) had recurrence at a median of 16.2 years (range, 3.6–51.9), and 2 developed metastatic PHEOs 20 years postoperatively. Meanwhile, in the present study, 27 patients (87.1%) had initial synchronous PHEOs. Of these, 23 bPHEOs were simultaneously and successfully removed, where the median size of the tumors was 3.8 cm, and 10 (43.5%) of them required steroid replacement at a median follow-up of 10.5 years (range, 1.8–23); none of them had recurrent and metastatic postoperatively (Tables 1, 3, 4). Nevertheless, 38.9% of the 18 patients underwent SB-LCSA or 60% of 5 patients underwent SB-OCSA, who respectively required steroid replacement, and the mean tumor size was (3.8 ± 1.7) or (5.1 ± 2.3) cm, with no significant difference (*P* = 0.397; *P* = 0.060). SB-LCSA can be safely performed and used for synchronous bPHEO surgery, which had the advantages of less blood loss and a shorter length of hospital stay than MB-OCSA (Table 4). Moreover, one case (P28), who had a recurrent PHEO (1.2 cm) on one side and developed a contralateral PHEO (5.3 cm), was also subjected to SB-LCSA (Table 1). However, reoperation may be more difficult than primary operations mainly due to adhesions, although laparoscopy can also be performed. SB-LCSA or SB-OCSA is technically safe and feasible (10, 12, 16–19); in particular, SB-LCSA can be considered a preferential choice in the surgical management for synchronous bPHEOs in MEN2, even for recurrent PHEOs.

As for whether the adrenal central vein should be preserved during the CSA, deliberately preserving it may be unnecessary based on our data and those of previous studies (16, 18, 22). However, avoiding excessive separation of the remnant adrenal gland from the adjacent space is important as the vascular bed adjacent to the remnant adrenal gland is integral to preserving its function. Corresponding evidence of the presence of successful adrenal autotransplantation was low (10, 16, 18, 22, 42). Furthermore, dissociating or ligating the central vein beforehand is not necessary. In general, the surrounding adrenal tissue can be separated at 0.5–1.0 cm from the PHEO using a harmonic scalpel. Especially when the preoperative CT/MRI scan demonstrated that the PHEO was large and the splenic/renal vessels were evidently compressed and deformed, it should be preferentially separated from the surrounding tissue vessels along the surface of the PHEO capsule, and the central vein could be ligated after the boundary was clear (Figures 2A,B). Meanwhile, once the specimen is removed, careful examination and preliminary assessment of a possible adequate disease-free margin around the PHEO needs to be performed.

## Conclusion

The integration of the clinical and molecular genetic diagnosis of MEN2 into routine practice can provide valuable information for establishing a precise treatment plan and procuring optimized guidance for long-term follow-up surveillance (9–11, 43–46). SB-LCSA with preserving adrenocortical function for treating synchronous bPHEOs in patients with MEN2 is safe and feasible and should be considered a prioritized surgical approach.

## Data Availability

The original contributions presented in the study are included in the article/Supplementary Material; further inquiries can be directed to the corresponding author.

## References

[1] Neumann HPH Tsoy U Bancos I Amodru V Walz MK Tirosh A Comparison of pheochromocytoma-specific morbidity and mortality among adults with bilateral pheochromocytomas undergoing total adrenalectomy vs cortical-sparing adrenalectomy. JAMA Netw Open. (2019) 2(8):e198898. 10.1001/jamanetworkopen.2019.8898 31397861 PMC6692838

[2] Castinetti F Qi XP Walz MK Maia AL Sansó G Peczkowska M Outcomes of adrenal-sparing surgery or total adrenalectomy in phaeochromocytoma associated with multiple endocrine neoplasia type 2: an international retrospective population-based study. Lancet Oncol. (2014) 15(6):648–55. 10.1016/S1470-2045(14)70154-8 24745698

[3] Castinetti F Waguespack SG Machens A Uchino S Hasse-Lazar K Sanso G Natural history, treatment, and long-term follow up of patients with multiple endocrine neoplasia type 2B: an international, multicentre, retrospective study. Lancet Diabetes Endocrinol. (2019) 7(3):213–20. 10.1016/S2213-8587(18)30336-X 30660595 PMC8132299

[4] Rossitti HM Söderkvist P Gimm O. Extent of surgery for phaeochromocytomas in the genomic era. Br J Surg. (2018) 105(2):e84–98. 10.1002/bjs.10744 29341163

[5] Bausch B Schiavi F Ni Y Welander J Patocs A Ngeow J Clinical characterization of the pheochromocytoma and paraganglioma susceptibility genes SDHA, TMEM127, MAX, and SDHAF2 for gene-informed prevention. JAMA Oncol. (2017) 3(9):1204–12. 10.1001/jamaoncol.2017.0223 28384794 PMC5824290

[6] Calsina B Currás-Freixes M Buffet A Pons T Contreras L Letón R Role of MDH2 pathogenic variant in pheochromocytoma and paraganglioma patients. Genet Med. (2018) 20(12):1652–62. 10.1038/s41436-018-0068-7 30008476 PMC7456538

[7] Gupta G. Pacak K, AACE Adrenal Scientific Committee. Precision medicine: an update on genotype/biochemical phenotype relationships in pheochromocytoma/paraganglioma patients. Endocr Pract. (2017) 23(6):690–704. 10.4158/EP161718.RA 28332883 PMC7470624

[8] Clark GR Sciacovelli M Gaude E Walsh DM Kirby G Simpson MA Germline FH mutations presenting with pheochromocytoma. J Clin Endocrinol Metab. (2014) 99(10):E2046–2050. 10.1210/jc.2014-1659 25004247

[9] Neumann HPH Young WF Jr Eng C. Pheochromocytoma and paraganglioma. N Engl J Med. (2019) 381(6):552–65. 10.1056/NEJMra1806651 31390501

[10] Kittah NE Gruber LM Bancos I Hamidi O Tamhane S Iñiguez-Ariza N Bilateral pheochromocytoma: clinical characteristics, treatment and longitudinal follow-up. Clin Endocrinol. (2020) 93(3):288–95. 10.1111/cen.14222 32410303

[11] Plouin PF Amar L Dekkers OM Fassnacht M Gimenez-Roqueplo AP Lenders JW European society of Endocrinology Clinical Practice Guideline for long-term follow-up of patients operated on for a phaeochromocytoma or paparaganglioma. Eur J Endocrinol. (2016) 174(5):G1–G10. 10.1530/EJE-16-0033 27048283

[12] Tuncel A Langenhuijsen J Erkan A Mikhaylikov T Arslan M Aslan Y Comparison of synchronous bilateral transperitoneal and posterior retroperitoneal laparoscopic adrenalectomy: results of a multicenter study. Surg Endosc. (2021) 35(3):1101–7. 10.1007/s00464-020-07474-y 32152673

[13] Wells SA Jr Asa SL Dralle H Elisei R Evans DB Gagel RF Revised American Thyroid Association guidelines for the management of medullary thyroid carcinoma. Thyroid. (2015) 25(6):567–610. 10.1089/thy.2014.0335 25810047 PMC4490627

[14] Lenders JW Duh QY Eisenhofer G Gimenez-Roqueplo AP Grebe SK Murad MH Pheochromocytoma and paraganglioma: an endocrine society clinical practice guideline. J Clin Endocrinol Metab. (2014) 99(6):1915–42. 10.1210/jc.2014-1498 24893135

[15] Osinga TE van den Eijnden MH Kema IP Kerstens MN Dullaart RP de Jong WH Unilateral and bilateral adrenalectomy for pheochromocytoma requires adjustment of urinary and plasma metanephrine reference ranges. J Clin Endocrinol Metab. (2013) 98(3):1076–83. 10.1210/jc.2012-3418 23365125

[16] Walz MK Alesina PF Wenger FA Koch JA Neumann HP Petersenn S Laparoscopic and retroperitoneoscopic treatment of pheochromocytomas and retroperitoneal paragangliomas: results of 161 tumors in 126 patients. World J Surg. (2006) 30(5):899–908. 10.1007/s00268-005-0373-6 16617419

[17] Biteman BR Randall JA Brody F. Laparoscopic bilateral cortical-sparing adrenalectomy for pheochromocytoma. Surg Endosc. (2016) 30(12):5622–3. 10.1007/s00464-016-4919-5 27177950

[18] Cheng SP Saunders BD Gauger PG Doherty GM. Laparoscopic partial adrenalectomy for bilateral pheochromocytomas. Ann Surg Oncol. (2008) 15(9):2506–8. 10.1245/s10434-008-0013-0 18618188

[19] Kumar S Choudhary GR Pushkarna A Prasad S Nanjappa B. Laparoscopic single-site synchronous bilateral cortex-preserving adrenalectomy using conventional trocars and instruments for large bilateral adrenal pheochromocytomas. Asian J Endosc Surg. (2014) 7(2):175–8. 10.1111/ases.12089 24754883

[20] Miccoli P Materazzi G Brauckhoff M Ambrosini CE Miccoli M Dralle H. No outcome differences between a laparoscopic and retroperitoneoscopic approach in synchronous bilateral adrenal surgery. World J Surg. (2011) 35(12):2698–702. 10.1007/s00268-011-1294-1 21976006

[21] Rodriguez JM Balsalobre M Ponce JL Ríos A Torregrosa NM Tebar J Pheochromocytoma in MEN 2A syndrome. Study of 54 patients. World J Surg. (2008) 32(11):2520–6. 10.1007/s00268-008-9734-2 18795243

[22] Brauckhoff M Stock K Stock S Lorenz K Sekulla C Brauckhoff K Limitations of intraoperative adrenal remnant volume measurement in patients undergoing subtotal adrenalectomy. World J Surg. (2008) 32(5):863–72. 10.1007/s00268-007-9402-y 18224482

[23] Qi XP Chen XL Ma JM Du ZF Fei J Yang CP RET proto-oncogene genetic screening of families with multiple endocrine neoplasia type 2 optimizes diagnostic and clinical management in China. Thyroid. (2012) 22(12):1257–65. 10.1089/thy.2012.0134 23210566

[24] Qi XP Zhao JQ Du ZF Yang RR Ma JM Fei J Prophylactic thyroidectomy for MEN 2-related medullary thyroid carcinoma based on predictive testing for RET proto-oncogene mutation and basal serum calcitonin in China. Eur J Surg Oncol. (2013) 39(9):1007–12. 10.1016/j.ejso.2013.06.015 23849459

[25] Chen S Li S Zhang J Zhang L Chen Y Wang L Preimplantation genetic diagnosis of multiple endocrine neoplasia type 2A using informative markers identified by targeted sequencing. Thyroid. (2018) 28(3):281–7. 10.1089/thy.2017.0200 29378479

[26] Li SY Ding YQ Si YL Ye MJ Xu CM Qi XP. 5P strategies for management of multiple endocrine neoplasia type 2: a paradigm of precision medicine. Front Endocrinol. (2020) 11:543246. 10.3389/fendo.2020.543246 PMC7531599 33071967

[27] Qi XP Jin BY Li PF Wang S Zhao YH Cao ZL RET S409y germline mutation and associated medullary thyroid carcinoma. Thyroid. (2019) 29(10):1447–56. 10.1089/thy.2018.0385 31364476

[28] Qi XP Zhao JQ Fang XD Lian BJ Li F Wang HH Spectrum of germline RET variants identified by targeted sequencing and associated multiple endocrine neoplasia type 2 susceptibility in China. BMC Cancer. (2021) 21(1):369. 10.1186/s12885-021-08116-9 33827484 PMC8028819

[29] Fang F Ding L He Q Liu M. Preoperative management of pheochromocytoma and paraganglioma. Front Endocrinol. (2020) 11:586795. 10.3389/fendo.2020.586795 PMC7551102 33117294

[30] Clavien PA Barkun J de Oliveira ML Vauthey JN Dindo D Schulick RD The Clavien–Dindo classification of surgical complications: five-year experience. Ann Surg. (2009) 250(2):187–96. 10.1097/SLA.0b013e3181b13ca2 19638912

[31] Flávio Rocha M Faramarzi-Roques R Tauzin-Fin P Vallee V Leitao de Vasconcelos PR Ballanger P. Laparoscopic surgery for pheochromocytoma. Eur Urol. (2004) 45(2):226–32. 10.1016/j.eururo.2003.09.016 14734011

[32] Gruber LM Hartman RP Thompson GB McKenzie TJ Lyden ML Dy BM Pheochromocytoma characteristics and behavior differ depending on method of discovery. J Clin Endocrinol Metab. (2019) 104(5):1386–93. 10.1210/jc.2018-01707 30462226

[33] Asari R Scheuba C Kaczirek K Niederle B. Estimated risk of pheochromocytoma recurrence after adrenal-sparing surgery in patients with multiple endocrine neoplasia type 2A. Arch Surg. (2006) 141(12):1199–205; discussion 1205. 10.1001/archsurg.141.12.1199 17178962

[34] Elisei R Alevizaki M Conte-Devolx B Frank-Raue K Leite V Williams GR. 2012 European thyroid association guidelines for genetic testing and its clinical consequences in medullary thyroid cancer. Eur Thyroid J. (2013) 1(4):216–31. 10.1159/000346174 24783025 PMC3821492

[35] Fu SQ Wang SY Chen Q Liu YT Li ZL Sun T. Laparoscopic versus open surgery for pheochromocytoma: a meta-analysis. BMC Surg. (2020) 20(1):167. 10.1186/s12893-020-00824-6 32711496 PMC7382066

[36] Chung HS Kim MS Yu HS Hwang EC Kim SO Oh KJ Laparoscopic adrenalectomy using the lateral retroperitoneal approach: is it a safe and feasible treatment option for pheochromocytomas larger than 6 cm? Int J Urol. (2018) 25(5):414–9. 10.1111/iju.13524 29478297

[37] Asher KP Gupta GN Boris RS Pinto PA Linehan WM Bratslavsky G. Robot-assisted laparoscopic partial adrenalectomy for pheochromocytoma: the National Cancer Institute technique. Eur Urol. (2011) 60(1):118–24. 10.1016/j.eururo.2011.03.046 21507561 PMC3109214

[38] Scholten A Valk GD Ulfman D Borel Rinkes IH Vriens MR. Unilateral subtotal adrenalectomy for pheochromocytoma in multiple endocrine neoplasia type 2 patients: a feasible surgical strategy. Ann Surg. (2011) 254(6):1022–7. 10.1097/SLA.0b013e318237480c 22107743

[39] Nagaraja V Eslick GD Edirimanne S. Recurrence and functional outcomes of partial adrenalectomy: a systematic review and meta-analysis. Int J Surg. (2015) 16(Pt A):7–13. 10.1016/j.ijsu.2015.01.015 25681039

[40] Gomella PT Sanford TH Pinto PA Bratslavsky G Metwalli AR Linehan WM Long-term functional and oncologic outcomes of partial adrenalectomy for pheochromocytoma. Urology. (2020) 140:85–90. 10.1016/j.urology.2020.02.015 32109495 PMC7255958

[41] Perysinakis I Aggeli C Kaltsas G Zografos GN. Adrenal-sparing surgery: current concepts on a theme from the past. Hormones. (2020 Sep) 19(3):317–27. 10.1007/s42000-020-00202-0 32388629

[42] Nockel P El Lakis M Gaitanidis A Yang L Merkel R Patel D Preoperative genetic testing in pheochromocytomas and paragangliomas influences the surgical approach and the extent of adrenal surgery. Surgery. (2018) 163(1):191–6. 10.1016/j.surg.2017.05.025 29126554 PMC5736453

[43] Ikeda Y Takami H Niimi M Kan S Sasaki Y Takayama J. Laparoscopic partial or cortical-sparing adrenalectomy by dividing the adrenal central vein. Surg Endosc. (2001) 15(7):747–50. 10.1007/s004640080112 11591982

[44] Buffet A Ben Aim L Leboulleux S Drui D Vezzosi D Libé R Positive impact of genetic test on the management and outcome of patients with paraganglioma and/or pheochromocytoma. J Clin Endocrinol Metab. (2019) 104(4):1109–18. 10.1210/jc.2018-02411 30698717

[45] Garcia-Carbonero R Matute Teresa F Mercader-Cidoncha E Mitjavila-Casanovas M Robledo M Tena I Multidisciplinary practice guidelines for the diagnosis, genetic counseling and treatment of pheochromocytomas and paragangliomas. Clin Transl Oncol. (2021) 23(10):1995–2019. 10.1007/s12094-021-02622-9 33959901 PMC8390422

[46] Muth A Crona J Gimm O Elmgren A Filipsson K Stenmark Askmalm M Genetic testing and surveillance guidelines in hereditary pheochromocytoma and paraganglioma. J Intern Med. (2019) 285(2):187–204. 10.1111/joim.12869 30536464

